# Developing Azeri aphasia screening test and preliminary validity and reliability

**Published:** 2016-10-07

**Authors:** Sousan Salehi, Ali Jahan, Najva Mousavi, Mazyar Hashemilar, Zohreh Razaghi, Maryam Moghadam-Salimi

**Affiliations:** 1 Department of Speech Therapy, School of Rehabilitation, Tehran University of Medical Sciences, Tehran, Iran; 2 Department of Speech Therapy, School of Rehabilitation, Tabriz University of Medical Sciences, Tabriz, Iran; 3 Department of Psychology, School of Education and Psychology, University of Tabriz, Tabriz, Iran; 4 Department of Neurology, School of Medicine, Tabriz University of Medical Sciences, Tabriz, Iran; 5 Endocrine Research Center, Tabriz University of Medical Sciences, Tabriz, Iran

**Keywords:** Screening Test, Aphasia, Azeri Language, Brain Injury, Iran

## Abstract

**Background:** As there is no standard aphasia screening tool for Azeri language yet, the aim of this study was to develop an aphasia screening test with acceptable validity and reliability.

**Methods:** The present study was conducted in two phases. In the first phase, by literature search, the screening test was designed and to obtain validity it was peer reviewed by expert panel. After collecting experts’ ratings and comments, appropriate modifications were applied. For test-retest reliability in the second phase, edited test was administered in 32 patients with brain injuries, then the retest was performed two weeks later.

**Results:** The developed test had eight subscales including: A) picture description, B) syntax, C) linguistic reasoning, D) descriptive naming, E) perception of minimal pairs, F) comprehensive vocabulary, G) expressive vocabulary, H) verbal fluency. Each section had five questions except verbal fluency which had 3 items. Content validity ratio (CVR) according to Lawshe’s approach, was 82% for the whole test. Intraclass correlation for all subscales were more than 0.8. Cronbach’s alpha coefficient for internal reliability was 0.901.

**Conclusion:** This aphasia screening test seems to have acceptable psychometric properties. This test can probably be used in clinical setting by specialists.

## Introduction

Aphasia is an acquired neurogenic language disorder.^1^ Stroke is the most common cause of aphasia.^2^ Aphasia incidence and prevalence is often estimated based on the incidence and prevalence of stroke.^3^ The incidence of ischemic stroke in Iranian population was reported to be 43.2 cases per 100,000 person-year. Frequency of aphasia among people who have experienced stroke is 33.3%.^4^

Aphasia screening measures are commonly concise. These tests are helpful in the early stages of recovery, when the patient still cannot complete long aphasia tests.^5^ There are several widely used tools for screening aphasia in other languages, some of them are being described as follows: 

Aphasia Language Performance Scale (ALPS): this tool includes four aspects of language (listening, speaking, reading, and writing) and each aspect has ten-item scale that their difficulty is gradually increased.^6^ ALPS is comprehensive in aspects of language but it has limitations for use in research projects.^7^

Acute Aphasia Screening Protocol (AASP): this test has four sub-scales including attention/orientation to communication (five items), auditory comprehension (15 items), expressive abilities (20 items), and conversational style (four items). Although it is short and easily can be done in clinical setting but it has subjective rating system in some sub-scales.^8^

Bedside Evaluation and Screening Test for Aphasia: this tool assesses language ability in three communicative modalities including auditory comprehension, speaking, and reading.^9^ It ignores writing which is an important modality in aphasia assessment in English language. 

Frenchay Aphasia Screening Test (FAST): it evaluates language in four language areas of comprehension, verbal expression, reading, and writing. Although this test is the most widely used screening tool,^8^ but it has limitations. It only applies visual materials, then any visual deficits such as neglect has adverse impact on patient’s score.^10^

Mississippi Aphasia Screening Test (MAST): MAST includes nine subscales comprising of naming, automatic speech, repetition, yes/no accuracy, object recognition, following verbal instructions, reading instructions, verbal fluency, and writing/spelling to dictation. It measures receptive and expressive language.^11^

Language Aphasia Screening Test (LAST): this test has two main indexes, receptive and expressive. Receptive index includes naming, repetition and automatic speech; expressive index includes recognition and verbal instructions.^12^

Mini-Mental State Examination (MMSE), Raven’s Colored Progressive Matrices (RCPM), and Sheffield Screening Test (SST) are short tests easily implemented, so can be suitable as screening tool^   13 ^ but none of them is comprehensive language assessment. In Iran, MAST has recently been translated with cross-cultural adaptation for Persian language.^   14^

The lack of valid and reliable test for clinical diagnosis and practice is a worldwide problem.^   15 ^ These tests are needed for early detection and intervention. Early detection of language impairments and synergy between intervention and neuroplasticity can maximize the benefits of treatment.^13^ 

Nevertheless, there is no Azeri Turkish aphasia screening test yet whereas we require a valid and reliable test in accordance with Azeri Turkish language structure and culture for clinical and research application. Azeri language in Iran do not have reading and writing, which can be considered as the most important feature of this language in developing the test. Then, the purpose of present study was developing the screening test of aphasia for Azeri speakers in Iranian population by minimizing limitations in other screening tests and obtaining preliminary validity and reliability as the first step toward standardization.

## Materials and Methods

***Development of the test***

The first section of this study was creating a new test for aphasia screening in Azeri language. Textbooks in linguistics and, language disorders and available screening tests for aphasia were reviewed. In general view, a suitable aphasia test should compromise content expression and comprehension (semantics), form (phonology, morphology and syntax) and pragmatic.^   2 ^ With respect to these guides and other literature, eight important domains of language were selected as follows:

1) Picture description (content production): this sub-test helps to assess the semantic and syntactic abilities by evaluating the retrieval of content and function words, and the arrangement of words in the sentence (grammar).^   16^

2) Syntax: syntactic processing is damaged in fluent aphasia.^   17 ^ Additionally, asyntactic comprehension is one of the high level processing problems in aphasia.^   2 ^ Asyntactic comprehension, negative forms, and prepositions are included in this part.

3) Verbal reasoning (pragmatic): in view of the fact that screening test should be sensitive to subtle deficit in cognition and communication, verbal reasoning was selected as part of the test. Verbal reasoning is higher level function that integrate several processes.^18^

4) Descriptive naming (comprehension): this complicated task is naming target items following verbal description. This task is sensitive to left lobe injuries.^19^ It needs language comprehension and word retrieval ability without visual processing involvement.

5) Minimal pairs (phonology): this task taps the auditory input processing without oral production. It can illustrate any problem in auditory analysis level.^20^  

6) Receptive vocabulary (single word comprehension): a basic task which assesses semantic input at single word level. Single word comprehension is not seriously disrupted in mild aphasia.^21^ Accordingly, Low frequency words were used in various semantic categories in the developed test to rise probability of error.

7) Expressive vocabulary (picture naming): it is reported that there is deficits in picture naming in all types of aphasia.^22^ Similar to receptive vocabulary, low frequency words in different semantic categories were included. 

8) Verbal fluency (semantic verbal fluency by naming animals): Verbal fluency refer to the number of words which is produced in one minute in specific semantic category; it can be included in aphasia assessment tests.^23^

As Iranian branch of Azeri language does not have writing form and is an oral language, then our screening test was not designed to include reading and writing parts. As mentioned before, the developed test had eight subscales, each subscale had five items except verbal fluency. 

Scoring system was 0 or 1 for each item (correct or incorrect answer); then range of score of each part was 0 to 5 except verbal fluency which had a score range of 0-3 (Table 1). 

***Validity***

At the second phase, content validity was determined. First, all the items were included in a questionnaire to verify their relevancy to the content and structure of the test. Then, the sheet with written explanation of our investigation was given to experts including nine experienced speech language pathologists and one linguist. Afterwards, according to expert’s opinions the test’s materials were modified or unacceptable items were deleted. Finally, ultimate form was obtained (appendix 1). Lawshe’s approach was used for determining content validity ratio (CVR) in quantitative way.^24^ 

***Reliability***

The reliability was obtained by test-retest and internal consistency evaluation. The test was administered in brain injury and stroke patients who were at risk of aphasia according to neurologist’s diagnosis, who were in the early stage of their injury or stroke. Participants included 32 brain injury and stroke patients, 11 female and 21 male with a mean age of 64 years [range: 43-86 and Standard deviation (SD) = 10.0[, who were referred to Imam Reza and Razi Hospitals in Tabriz, Iran. All of them were under medication and were native Azeri speakers. Informed consent was taken according to ethical committee of Tabriz University of Medical Sciences. Participants were assessed for the second time after two weeks.

## Results

The patients’ scores are shown in each subscale in table 2. Content validity coefficients were calculated for each item in subscales; there were totally 38 items. Content validity coefficient was 40% for four items, 62% for eight items, 80% for seventeen items and 100% for nine items. Since the acceptable CVR is 62%, four items which had CVR less than 62% were modified. Then, the average of the rest of the items was calculated as the content validity indicator. Thus, the whole content validity coefficient was obtained as 82%.

In the second phase of the study, intraclass correlation coefficient (ICC) was calculated for each subscale. Pearson's correlation coefficients of subscales are also presented in table 3.

As for verbal fluency, it was analyzed by the Spearmen's correlation coefficient. The coefficient of 0.899 was obtained for verbal fluency which is well above 0.7. The ICC for this subscale was 0.928.

High Pearson's correlation coefficient between test-retest scores as well as high ICC (above 0.75) showed the acceptable level of test-retest reliability.^   25 ^ Cronbach's alpha was used to determine internal consistency of the test. For eight subscales, Cronbach' alpha was obtained as 0.91, indicating a high reliability for Azeri aphasia screening test.

## Discussion

An attempt was made to develop a valid and reliable test which encompasses important language domains in multimodality. In descriptive naming, verbal reasoning and verbal fluency items, the stimulus was only auditory and it is useful for patients who has visual deficit. 

**Table 1:** The subscales and items

**Test’s ** **subscales**	**Picture description ** **(content production)**	**Syntax** **(comprehension)**	**Verbal reasoning ** **(pragmatic)**	**Descriptive ** **naming**	**Minimal pairs ** **(phonology)**	**Receptive ** **vocabulary**	**Expressive ** **vocabulary**	**Verbal fluency** **animal’s names**
Item 1	Father plays with toys	The large glass which is broken	Watermelon skin is red and its inside is green. Is it right?	Wash hands with what?	Dog, rope	Barrel	Flag	No name
Item 2	Mother saw it	The broken flower which is under the table	We can brush our teeth with spoon instead of tooth brush. Is it right?	Children draw with what?	Tongue, teeth	Loudspeaker	Lantern	5 names
Item 3	Son cooks the food	Father of kids who do not say goodbye.	It is possible to put the pen in the pot. Is it right?	What is the name of person who drives airplane?	Park, pitcher	Urceolate(bell)	Funnel	More than 5 names
Item 4	Daughter read the newspaper	The cat that looks the boy	We have breakfast between lunch and dinner. Is it right?	What does the fan exactly do?	King, scarf	Boat	Feather	
Item 5	It is expected that the patient point to relationship between them.	The girls who do not look the boy	It’s snowing in the summer. Is it right?	What does the cat eat?	Stone, head	Button	Scale	

**Table 2:** Descriptive statistics of scores in various subscales of test

**Descriptive statistics of scores**	**Minimum**	**Maximum**	**Mean ± SD**
**Subscales**			
Picture description (content production)	0	4	1.85 ± 1.79
Syntax (comprehension)	0	5	2.62 ± 1.63
Verbal reasoning (pragmatics)	0	5	3.32 ± 1.82
Descriptive naming	0	5	2.45 ± 2.19
Minimal pairs (phonology)	0	5	2.77 ± 1.74
Receptive vocabulary	0	5	3.72 ± 1.57
Expressive vocabulary	0	5	2.35 ± 1.70
Verbal fluency Animal’s names	0	2	0.87 ± 0.82

SD: Standard deviation

**Table 3:** Pearson correlation and intraclass correlation coefficient (ICC) values

**Parts of test**	**Pearson's correlation coefficient**	**ICC**
Picture description	0.787	0.88
Syntax	0.823	0.897
Verbal reasoning	0.832	0.908
Descriptive naming	0.936	0.964
Minimal pairs	0.817	0.892
Receptive vocabulary	0.835	0.91
Expressive vocabulary	0.829	0.906
Total score	0.936	0.966

ICC: Intraclass correlation coefficient

In other items, stimulus was visual and it is suitable for patients who has auditory deficit. As mentioned, all language domains including phonology, syntax, semantic and pragmatic were presented in various items. Patient response had two main categories, expressive and receptive, which is similar to screening tests like LAST and FAST. The developed test did not have reading and writing subscales, because of special Azeri Turkish feature, which is a verbal language. The highest score in intraclass correlation in eight subscale was descriptive naming, then verbal fluency, receptive vocabulary, and verbal reasoning. All of these items were present in auditory modality. After these four items, there was expressive vocabulary, picture description, syntax, and finally minimal pairs. These items were presented visually. 

Inter-item correlation was utilized to specify the internal reliability. It was the highest in all items for descriptive naming item, then verbal fluency, verbal reasoning and receptive vocabulary, expressive vocabulary, syntax, minimal pairs, and picture description, respectively. This is nearly similar to intraclass correlation. Thus the first four items that all of them were auditory (descriptive naming, verbal fluency, verbal reasoning, and receptive vocabulary) were appropriate for aphasia screening. In the next four items, minimal pairs and picture description, were not proper to this evaluation. Minimal pairs was not in reviewed aphasia screening tests for assessing phonology. However, the expressive vocabulary (picture naming) and syntax sub-tests were apparently more suitable for screening aphasia.

Test-retest reliability was a common approach in determining test reliability. The reliability coefficient for ALPS was reported from 0.83 to 0.94 for aphasic patients; retest was from 3 to 5 weeks after the beginning test.^   26 ^ For AASP, another aphasia screening test, test-retest greater than 0.7 was reported.^        27 ^ Reliability coefficient ranged from 0.93 to 0.99 for all subscales of Bedsides Evaluation and Screening Test of Aphasia.^9^ Criterion validity and test-retest reliability to FAST was reported 0.96 and 0.97, respectively.^   10 ^ There was not any report on reliability of MAST.^   12 ^ Interrater reliability was obtained for LAST instead of test-retest reliability, thus we cannot compare it with our results. Our aphasia screening test had a 0.93 test-retest reliability, suggesting that the developed test has high temporal stability. It seems acceptable compared to the reliability of other screening test. 

ICC of LAST was 0.96, indicating good internal validity and Chronbach’s alpha was 0.88, indicating good internal cohesion.^   13 ^ Total CVR of the developed test was 0.82 according to Lawshe’s content validity table which is acceptable compared to other tests (> 0.62). Therefore, the content validity of this test seems to be appropriate. In this study, we did not calculate criterion validity. 

## Conclusion

The results of this preliminary study suggested that the developed aphasia screening test for Turkish Azeri language had similar validity and reliability to other screening test in other languages. It seems this test has acceptable psychometric values and it can be used in clinic and research for early diagnosis of aphasia. For further investigation, it is recommended that other types of validity and reliability should be calculated and the test also can be performed in normal population to obtain norm scores of the test.

## Notes:

**How to cite this article:** Salehi S, Jahan A, Mousavi N, Hashemilar M, Razaghi Z, Moghadam-Salimi M. Developing Azeri aphasia screening test and preliminary validity and reliability. Iran J Neurol 2016; 15(4): 183-8.
